# Predictive value of CHA_2_DS_2_VASC score for contrast-induced nephropathy after primary percutaneous coronary intervention for patients presenting with acute ST-segment elevation myocardial infarction

**DOI:** 10.1186/s43044-023-00378-x

**Published:** 2023-06-26

**Authors:** Ahmad Samir, Wafik Gabra, Hossam Alhossary, Sameh Bakhoum

**Affiliations:** 1 Cardiology Department, Faculty of Medicine, Cairo University, Cairo, Egypt; 2 Cardiology Department, National Heart Institute, Cairo, Egypt

**Keywords:** CHA_2_DS_2_VAS_C_, Contrast-induced nephropathy CIN, ST-elevation myocardial infraction STEMI, Mehran

## Abstract

**Background:**

ST-elevation myocardial infarction (STEMI) patients undergoing primary percutaneous coronary intervention (pPCI) are at increased risk for contrast-induced nephropathy (CIN) than elective PCI procedures. Routine calculation of Mehran’s score is limited by its complexity and difficulty to memorize. This study evaluated CHA_2_DS_2_-VASc score predictive utility for CIN in STEMI patients before pPCI.

**Results:**

Consecutive 500 acute STEMI patients presenting to two Egyptian pPCI centers were recruited. Exclusion criteria included cardiogenic shock or known severe renal impairment (baseline serum creatinine ≥ 3 mg/dL) or current or previous indication of hemodialysis. CHA_2_DS_2_VAS_C_ score_,_ Mehran’s score, baseline estimated glomerular filtration rate (eGFR), contrast media volume (CMV) and CMV/eGFR ratio were collected for all patients. Post-pPCI CIN (defined as 0.5 mg/dL absolute increase or 25% relative increase of serum creatinine from baseline) and predictive accuracy of CHA_2_DS_2_VAS_C_ and Mehran’s scores were evaluated. CIN occurred in 35 (7%) of the study group. Values of CHA_2_DS_2_VAS_C_ score_,_ Mehran’s score, baseline eGFR, CMV and CMV/eGFR ratio were significantly higher in those who developed CIN compared to those who did not. CHA_2_DS_2_VAS_C_ score_,_ Mehran’s score and CMV/eGFR were found to be independent predictors for CIN (*P* < 0.001 for all). ROC curve analysis revealed that CHA_2_DS_2_VAS_C_ ≥ 4 had a superb predictive ability, comparable to Mehran’s score, for post-pPCI CIN.

**Conclusions:**

Being practical, easily memorizable and applicable before proceeding to pPCI, routine CHA_2_DS_2_VAS_C_ score calculation in STEMI patients can effectively predict CIN risk and guide preventive and/or therapeutic interventions.

## Background

Contrast-induced nephropathy (CIN), or acute kidney injury (AKI) following exposure to iodinated contrast media, represents a worrying complication following coronary angiography and/or percutaneous coronary intervention (PCI) [1]. The most widely used thresholds for CIN diagnosis are either increase in serum creatinine by 0.5 mg/dL (44 μmol/L) or by more than 25% from baseline level, with incidence peaking from days 2 to 5 after the contrast exposure [2–4]. Reported incidence of CIN ranges from 6 to 25% according to the characteristics of the studied population and the clinical scenario. CIN is significantly associated with worse short- and long-term outcomes including renal failure and death [1, 2, 4].

Thereby, prediction of CIN risk is critical, particularly if it can be identified early pre-procedure, to provide intensified preventive measures for those at higher risk, before-, during- and after contrast exposure.

Mehran’s score is among the most sensitive and most widely used models for predicting CIN- and hemodialysis risks after coronary angiography and/or PCI [5]. However, its systematic use may be limited because of its complexity and difficulty to memorize rendering its use very selective (when high CIN risk is clinically suspected).

ST-segment elevation myocardial infarction (STEMI) represents one of the most serious presentations of atherosclerotic cardiovascular disease (ASCVD) with average in-hospital and 1-year mortality rates of 6% and 10%, respectively [6]. Primary PCI (pPCI) to restore patency of the infarct-related artery (IRA) is the recommended management strategy for acute STEMI patients, particularly if it can be performed within the allowable time delay. Despite inevitable contrast exposure, timely reperfusion by pPCI is prioritized over awaiting any laboratory results (including cardiac biomarkers or kidney function tests) [6].

Emergent PCI—such as in STEMI settings—inherently carries a higher risk for CIN compared elective PCI procedures [7]. Additionally, the majority of STEMI patients proceed to pPCI before knowing their kidney functions, frequently have a higher burden of the factors already proven to be associated with increased CIN risk (hypertension, diabetes, more likely to have acute heart failure, cardiogenic shock, require intra-aortic balloon pump (IABP) and more likely to be deprived from oral hydration due to the severe pain and agony in the hours prior to pPCI) [3, 5, 7].

Thereby, it would be extremely useful in STEMI management to have a practical and easily memorizable tool, allowing systematic use in emergency departments before proceeding to pPCI, while promptly providing the needed information (i.e., independent from laboratory workup or contrast volume after the procedure ends). Such a tool can guide critical procedural/technical optimizations for those identified at high CIN risk, like selection of nonionic over ionic contrast products or avoidance of ad-hoc PCI to severe non-culprit lesions [8]. While CHA_2_DS_2_VASc score was originally developed to evaluate thromboembolic risk in patients with non-valvular atrial fibrillation (AF), it was found to have a very sensitive prognostic utility in many other cardiovascular (CV) conditions [9–12].

In the current study, predictive utility of CHA_2_DS_2_VASc score for post-pPCI CIN in acute STEMI patients was assessed and contrasted against Mehran’s score.

## Methods

### Study design

This is an observational prospective cohort study that recruited consecutive 500 eligible acute STEMI patients. The study cohort were recruited from two tertiary pPCI centers in Egypt, (Cairo University hospitals and Egyptian National Heart Institute) through April 2021 to March 2022. The study protocol was approved by ethics committees in both participating centers and was registered on clinicaltrials.gov [NCT04730778].

Inclusion criteria were; age between 18 and 80 years old, established diagnosis of STEMI according to 4^th^ universal definition of myocardial infarction [13], presentation within 24 hours from symptoms onset, completion of successful pPCI (restoration of thrombolysis in myocardial infarction (TIMI) flow grade ≥ 2 in the IRA) and acceptance to participate in the study via a written informed consent. Exclusion criteria were; patients presenting with cardiogenic shock; having received thrombolysis prior to referral, or having history of significant kidney disease including current or prior indication for hemodialysis, or previous kidney function assessments denoting serum creatinine > 3 mg/dL or grade ≥ 3a chronic kidney disease (CKD) defined as estimated glomerular filtration rate (eGFR) of < 45 mL/min/1.73 m^2^) [14].

### Study protocol

While preparing eligible STEMI patients for transfer to pPCI, they were subjected to targeted history taking, clinical examination, 12-lead electrocardiography (ECG), blood sampling for routine laboratory work and a brief bedside echocardiography, provided that none of these is delaying revascularization.

Acute STEMI diagnosis was established when a patient presented with ischemic symptoms (anginal chest pain or equivalents) with new ST-segment elevation of ≥ 1mm in at least 2 contiguous leads. Diagnostic threshold for ST-segment elevation in leads V2 and V3 is ≥ 1.5 mm in women, ≥ 2 mm in men above the age of 40 years and ≥ 2.5 mm in men < 40 years. Alternatively, anterior STEMI was diagnosed if the same clinical scenario was associated with new (or presumable new) left bundle branch block, while posterior STEMI was diagnosed with tall R wave with ST depression in V1-V3 subsequently confirmed by ST elevation in posterior leads (V7-V9) [6, 13].

For the sake of prioritizing reperfusion, results for laboratory work (including serum creatinine) were allowed to be reviewed after pPCI completion. Estimated GFR was calculated by the modification of diet in renal diseases (MDRD) 4-variable formula [15].

Demographic characteristics, clinical evaluation, time delays, ECG and echocardiographic findings were tabulated by medical record numbers anonymized from personal identifiers. Hypertension was defined as consistent blood pressure > 140/90 mmHg or being controlled on antihypertensive therapy. Diabetes was defined as glycated hemoglobin > 6.5% or being controlled on antidiabetic therapy. Dyslipidemia was defined as low-density lipoprotein cholesterol (LDL-C) > 130 mg/dL and/or high-density lipoprotein (HDL-C) < 40 mg/dL [16].

Coronary angiogram and pPCI were performed according to standard techniques for all patients followed by guidelines directed medical therapy [6]. According to standard practice in both recruiting centers, all patients received nonionic low-osmolar iodine contrast (Ultravest-370, Schering, Germany; osmolarity 880 mOsm/kg, iodine content 370mg/mL) and culprit vessel was managed by implanting contemporary generation of drug eluting stents (DES). After pPCI, procedural data and baseline laboratory workup were tabulated as well. Technical success of pPCI was evaluated by the final TIMI flow grade ≥ 2 in the culprit vessel [17].

Post-pPCI serum creatinine levels were assessed daily for at least 2 postprocedural days (the minimum hospital stay for uneventful cases). Patients showing serum creatinine rise of ≥ 0.2 mg/dL or ≥ 10% relative increase from baseline (pre-procedure) were subjected to bi-daily testing and longer than 2 days of follow-up. CIN was defined as either absolute rise of serum creatinine level ≥ 0.5 mg/dL or relative rise of ≥ 25% from baseline (pre-procedure) values, through a minimum of 2 days and up to 7 days after the PCI [3]. The subgroup of patients who developed CIN are contrasted to the other group (No-CIN) to identify characteristics, risk factors and procedure-related predictors.

### Systematic calculation of risk scores

CHA_2_DS_2_VASc [18, 19] and Mehran’s score [5] were systematically calculated for all study participants as demonstrated in Table 1. Being dependent on basic clinical features, CHA_2_DS_2_VASc was calculated before to the procedure, while Mehran’s score was completed after procedure ends to integrate the awaited laboratory results (hematocrit and eGFR) and the total amount of contrast used. Contrast media volume/eGFR (CMV/eGFR) ratio was calculated for all patients at the end of the procedure as well.

**Table 1:** CHA_2_DS_2_VASc [18, 19] and Mehran’s score [5] definitions and calculation

Variable	Score	Description			
**CHA**_**2**_**DS**_**2**_**VASc score**					
C	1	Congestive heart failure or LVEF ≤ 40%			
H	1	Hypertension; BP consistently > 140/90 mmHg, or controlled on medications			
A_2_	2	Age ≥ 75 years			
D	1	Diabetes mellitus, defined as HbA1c ≥ 6.5 gm/dL or controlled on medications			
S_2_	2	Stroke, TIA or systemic thromboembolism			
V	1	Vascular disease, defined as prior MI, peripheral arterial disease or aortic plaque			
A	1	Age 65–74 years			
Sc	1	Sex category (female)*			
**Mehran’s risk score**					
Hypotension	5	Systolic BP < 80 mmHg or > 1 h on inotropic support			
Heart failure	5	NYHA class III/IV or recent pulmonary edema			
IABP	5	Intra-aortic balloon pump therapy			
Age	4	If > 75 years			
Anemia	3	HCT < 0.39 for males, < 0.36 for females			
Diabetes	3	Diabetes mellitus affecting blood sugar levels			
eGFR	2	For eGFR 60–40 mL/min			
	4	For eGFR 40–20 mL/min			
	6	For eGFR < 20 mL/min			
Contrast volume		1 point for every 100 mL			
	Total Mehran’s score	< 5	6–10	11–16	> 16
	CIN risk	7.5%	14%	26.1%	57.3%
	Dialysis risk	0.04%	0.12%	1.09%	12.6%

*LVEF* left ventricular ejection fraction, *TIA* transient ischemic attack, *BP* bloop pressure, *CIN* contrast-induced nephropathy, *eGFR* estimated glomerular filtration rate, *HCT* hematocrit value, *MI* myocardial infarction, *NYHA* New York Heart Failure Association

*Female sex counted as 1 point in the presence of other risk factors

### Sample size and statistical analysis

Sample size was calculated using G*Power software version 3.1.9.4 for MS Windows, (Franz Faul, Kiel University, Germany). CIN incidence was postulated to be 5% in this protocol-eligible STEMI patients. Planning to perform multivariate logistic regression to detect independent predictors for CIN in the subgroup who will develop it, considering type 1 error as 0.05 and a study power of 95%, a sample size of 500 eligible STEMI patients was planned.

Data devoid from personal identifiers were subjected to statistical analysis using IBM SPSS (Statistical Package for the Social Science; IBM Corp, Armonk, NY, USA) release 22 for Microsoft Windows and MedCalc Statistical Software version 14.10.2 (MedCalc Software bvba, Ostend, Belgium).

Categorical variables were expressed as frequencies and percentages, while continuous variables were expressed as mean ± standard deviation (± SD) or median and inter-quartile range as appropriate. Comparison of continuous variables between the CIN and No-CIN groups was done using Student's t test for independent samples or Mann–Whitney U test according to normality of data distribution. For comparing categorical data, Chi-square (*χ*^2^) test was performed or alternatively exact test when the expected frequency is less than 5. Correlations between various variables were tested using Pearson and Spearman-rank correlation equations for normally distributed and non-normally distributed variables, respectively. Univariate and multivariate regression tests were conducted for the appropriate individual variables of both CHA_2_DS_2_VASc- and Mehran’s scores to evaluate their predictive utility for CIN. Vascular disease (V) was excluded from the analyses having all the study patients presenting with STEMI and qualifying a score of (1) in this point. Receiver operator characteristic (ROC) analysis was used to determine the optimum cutoff value to predict CIN. Accuracy was represented in sensitivity, specificity, positive predictive value (PPV), negative predictive value (NPV) and overall accuracy. Two-sided *P* value ≤ 0.05 was considered statistically significant.

## Results

This study recruited 500 consecutive eligible STEMI patients who presented for pPCI over a period of 1 year (April 2021 to March 2022). CIN diagnosis was established in 35 patients (7%) of the study group. Baseline characteristics of the whole study group, those who developed CIN (CIN group) compared to those who did not (No-CIN group), are demonstrated in Table 2.

**Table 2:** Baseline characteristics of the whole study group, the CIN and the No-CIN groups

	All study group (*n* = 500)	CIN group (*n* = 35)	No-CIN group (*n* = 465)	*P* value
Age (years)	54.58 ± 10.23	65.23 ± 7.24	53.77 ± 9.97	< 0.001
Male gender	425 (85%)	27 (77.14%)	398 (85.59%)	0.215
Diabetes mellitus	226 (45.2%)	23 (65.71%)	203 (43.66%)	0.013
Hypertension	284 (56.8%)	26 (74.29%)	258 (55.48%)	0.034
Dyslipidemia	460 (92.00%)	35 (100%)	425 (91.4%)	0.098
Prior stroke or TIA	10 (2.00%)	5 (14.29%)	5 (1.08%)	< 0.001
Abnormal kidney functions*	44 (8.80%)	11 (31.43%)	53 (11.4%)	0.002
Smoking	406 (81.20%)	27 (77.14%)	379 (81.51%)	0.505
NYHA III/IV	36 (7.20%)	11(31.43%)	25 (5.37%)	< 0.001
Killip class I	421 (84.2%)	16 (45.71%)	405 (87.1%)	< 0.001
Killip class II	56 (11.2%)	6 (17.14%)	50 (10.75%)	0.263
Killip class III	23 (4.6%)	13 (37.14%)	10 (2.15%)	< 0.001
Anterior STEMI	384 (76.80%)	29 (82.86%)	355 (76.34%)	0.456
Lateral STEMI	20 (4%)	0 (0%)	20 (4.30%)	
Inferior STEMI	96 (19.20%)	6 (17.14%)	90 (19.3%)	
Final TIMI flow II	6 (1.20%)	0 (0%)	6 (1.29%)	1.000
Final TIMI flow III	494 (98.80%)	35 (100%)	459 (98.71%)	
Total ischemic time (min)^$^	76.37 ± 30.38	116.57 ± 50.5	73.74 ± 29.33	< 0.001
CMV	213.26 ± 45.90	262.57 ± 48.77	209.55 ± 43.52	0.001
CMV/eGFR ratio	2.45 ± 0.97	3.92 ± 1.77	2.34 ± 0.78	< 0.001
Hematocrit	43.11 ± 5.94	42.81 ± 5.82	44.07 ± 6	0.76
RBG	144.66 ± 37.20	163.86 ± 43.57	143 ± 36.31	0.09
Baseline serum creatinine	0.91 ± 0.20	1.07 ± 0.33	0.89 ± 0.18	< 0.001
eGFR (mL/min/1/73 m^2^)	88.45 ± 24	69.66 ± 15.91	89.86 ± 23.91	< 0.001
LVEF	50.60 ± 8.59	48.83 ± 7.12	50.73 ± 8.67	0.169
Moderate/severe MR	18 (3.6%)	4 (11.43%)	14 (3.01%)	0.035
CHA2DS2 VASC score	2 [1, 2]	4 [4, 5]	1 [1, 2]	< 0.001
Mehran score	3 [2–5]	10 [10–14]	2 [2–5]	< 0.001

Continuous data are expressed as mean ± (standard deviation) or median [inter-quartile range] as appropriate, while categorical data are expressed as frequency (percentage)

*CIN* contrast-induced nephropathy, *CMV* contrast media volume, *eGFR* estimated glomerular filtration rate, *LVEF* left ventricular ejection fraction, *NYHA* New York heart failure, *RBG*,random blood glucose, *TIA* transient ischemic attack, *TIMI*,thrombolysis in myocardial infarction

^*^Baseline serum creatinine above the normal range (1.2 mg/dL), yet eGFR remains > 40 mL/min/1.73 m^2^

^$^Representing time from symptoms onset to time of restoring flow in the culprit artery

Compared to the No-CIN population, patients of the CIN group were characterized by significantly older age (65.23 ± 7.24 vs. 53.77 ± 9.97, *P *< 0.001), higher rates of diabetes (23 (65.71%) vs. 203 (43.66%), *P *= 0.013), hypertension (26 (74.29%) vs. 258 (55.48%) *P *= 0.034) and history of prior stroke/TIA (5 (14.29%) vs. 5 (1.08%), *P* < 0.001). Gender, dyslipidemia and smoking were found to be comparable across groups.

Presentation with heart failure NYHA class III/IV and/or Killip class ≥ III was more prevalent in those who developed CIN compared to those who did not, (11 (31.43%) vs. 25 (5.37%) and 13 (37.14%) vs. 10 (2.15%), respectively, *P* < 0.001 for both). Total ischemic time (symptoms onset to reperfusion) was significantly longer in the CIN group compared to the No-CIN group (116.57 ± 50.5 vs. 76.37 ± 30.38 minutes, *P* < 0.001); however, type of STEMI (anterior, lateral vs. inferior) did not differ across the groups.

Concerning baseline laboratory results, hematocrit and random blood glucose values were found comparable. However, serum creatinine was significantly higher and eGFR was significantly lower in the CIN group compared to the No-CIN group (1.07 ± 0.33 vs. 0.89 ± 0.18, and 89.86 ± 23.91 vs. 69.66 ± 15.91, respectively, *P* < 0.001 for both). In echocardiographic assessment, CIN group had a significantly higher rate of ≥ moderate mitral regurgitation (4 (11.43%) vs. 14 (3.01%), *P *= 0.035), but a statistically nonsignificant lower LVEF values (48.83 ± 7.12 vs. 50.73 ± 8.67, *P *= 0.169) compared to the No-CIN group.

Regarding the pPCI, all patients underwent successful reperfusion with restoration of IRA patency. TIMI flow grade was comparable between the 2 groups. Compared to the No-CIN group, patients who developed CIN received significantly larger CMV (262.57 ± 48.77 vs. 209.55± 43.52, *P* < 0.001), and had a significantly higher CMV/eGFR ratio (3.92 ± 1.77 vs. 2.34 ± 0.78, *P* < 0.001).

CHA_2_DS_2_VASc score calculated at presentation showed a significant difference with higher scores in the CIN- compared to the No-CIN group, (median [inter-quartile range (IQR): 4 [4–5] vs. 1 [1–2], *P* < 0.001). Similarly, Mehran’s score calculated after the end of the procedure and availability of the laboratory results showed significantly higher values for the CIN- compared to the No-CIN group (10 [10–14] vs. 2 [2–5], *P* < 0.001).

In univariate regression analysis for the relevant components of CHA_2_DS_2_VASc score, it was found that CHF (odds ratio (OR): 4.49, *P *< 0.001), hypertension (OR 3.88, *P *= 0.003), diabetes (OR 2.74, *P *= 0.014), age ≥ 65 years (OR 23.97, *P *< 0.001), age ≥ 75 years (OR 11.87, *P *< 0.001) and stroke/TIA (OR 15.33, *P *< 0.001) were significant predictors for CIN, while female gender was not. In multivariate regression model, CHF, diabetes, age 65 to 74 years and prior stroke/TIA were found to be independent predictors for post-pPCI CIN.

Similarly, in univariate regression analysis for the relevant components of Mehran’s score, hypotension (OR 26.86, *P *< 0.001), CHF (OR 4.49, *P *< 0.001), age ≥ 75 years (OR 11.87, *P *< 0.001), diabetes (OR 2.74, *P *= 0.014) and contrast volume score of ≥ 3 (OR 8.72, *P *< 0.001) were found to be significant predictors for CIN. While in the multivariate regression model, hypotension, CHF and CMV score of ≥ 3 were independent predictors for CIN.

When expressed as a continuous variable, CMV (in mL) was a predictor for CIN in univariate regression yet with a marginal added risk (OR of 1.03 and 95% confidence interval (95%CI) of (1.02-1.04), while this relation was insignificant when tested in multivariate regression models. However, when indexed to the patient’s eGFR, the CMV/eGFR ratio was found to be a significant predictor in univariate and multivariate regression models with an OR (95% CI) of 3.42 (2.89–5.11) and 2.45 (1.49–4.03), respectively, *P *< 0.001 for both. Table 3 demonstrates the relations of CHA_2_DS_2_VASc, Mehran’s score and their individual components to CIN occurrence.

**Table 3:** Univariate and multivariate regression analysis for CAHADSVASC-, Mehran’s score components and CMV/eGFR ratio to predict CIN

	Univariate regression analysis		Multivariate regression analysis	
	OR (95% CI)	*P* value	OR (95% CI)	*P* value
CHF	4.49 (2.14–9.45)	< 0.001	4.32 (1.58–11.81)	0.004
Hypertension	0.25 (0.029– 2.18)	0.211	–	–
Diabetes	2.74 (1.37–5.5)	0.014	3.1 (1.29–7.48)	0.012
Age from 65 to 74 years	23.97 (10.63–54.05)	< 0.001	61.07 (19.38–192.47)	< 0.001
Age ≥ 75 years	11.87 (3.03–46.44)	< 0.001	–	–
Female gender	1.76 (0.77–4.04)	0.182	–	–
Stroke/TIA	15.33 (4.21–55.89)	< 0.001	92.52 (15.55–550.64)	< 0.001
Hypotension	26.86 (10.62–68.06)	< 0.001	85.34 (11.41–637.9)	< 0.001
CHF	4.49 (2.14–9.45)	< 0.001	59 (61.98—5044)	< 0.001
Age ≥ 75 years	11.87 (3.03–46.44)	< 0.001	–	–
Diabetes	2.47 (1.2–5.09)	0.014	–	–
CMV (≥ 3)	8.72 (3.74–20.36)	< 0.001	1.06 (1.04–1.09)	< 0.001
CMV (mL)	1.03 (1.02–1.04)	< 0.001	1.01 (0.99–1.02)	0.053
CMV/eGFR ratio	3.42 (2.89–5.11)	< 0.001	2.45 (1.49–4.03)	< 0.001

95% CI, 95% confidence interval; CHF, congestive heart failure or reduced left ventricular ejection fraction, CMV, contrast media volume; eGFR, estimated GFR (by MDRD-4); OR, odds ratio; TIA, transient ischemic attack

**Fig. 1:**
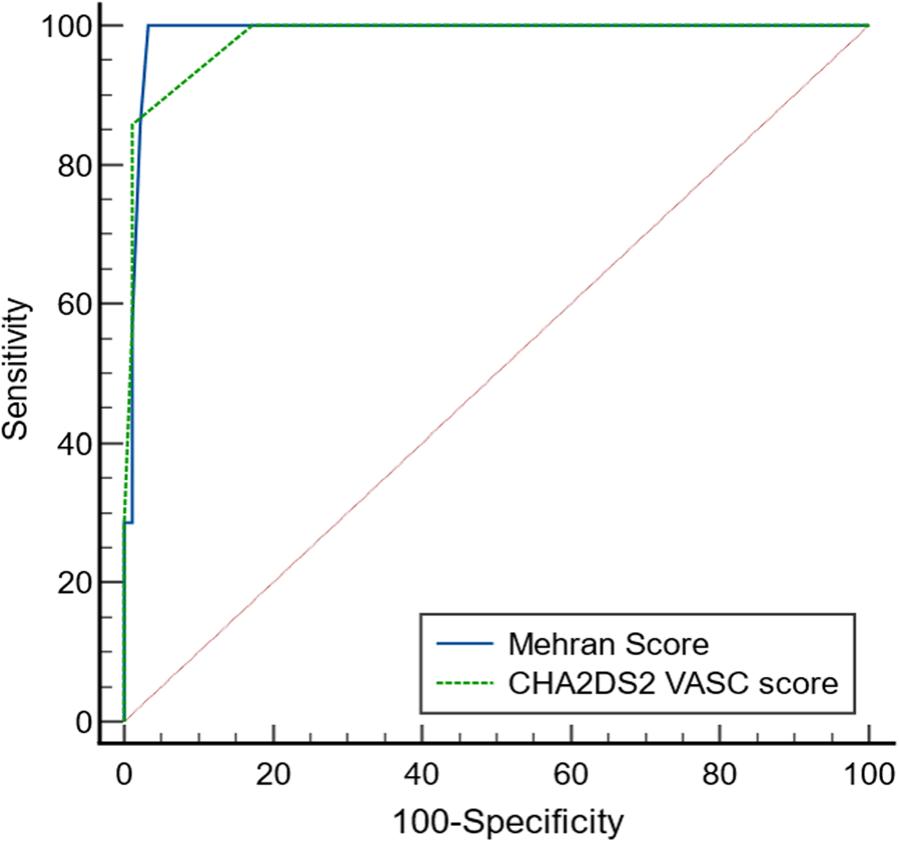
ROC curve analysis of CHA2DS2VASC and Mehran scores for prediction of CIN in the study participants

ROC curve analysis revealed that CHA_2_DS_2_VASc score had an excellent predictive ability to the occurrence of CIN (AUC: 0.982, *P *< 0.001). At a cutoff value of ≥ 4 CHA_2_DS_2_VASc has a sensitivity of 85.74%, specificity of 98.92%, PPV of 85.7% and NPV of 98.9%. Similarly, Mehran’s score proved to be an excellent predictor for CIN (AUC: 0.988, *P *< 0.001). At a cutoff value of ≥ 7, Mehran’s score had a sensitivity of 100%, specificity of 96.77%, PPV of 70% and NPV of 100%. Comparison of the 2 ROC curves revealed no significant difference between CHA_2_DS_2_VASc- and Mehran’s score in the prediction of CIN after pPCI. Detailed ROC curve analysis and comparison are demonstrated in Table 4 and Fig. 1, respectively.

**Table 4:** Diagnostic accuracy of CHA_2_DS_2_VASc- and Mehran’s scores for prediction of CIN in the study participants

	Cutoff	AUC	Sens	Spec	PPV	NPV	*P* value
CHA_2_DS_2_VASc score	≥ 4	0.982	85.74	98.92	85.7	98.9	< 0.001
Mehran score	≥ 7	0.988	100	96.77	70	100	< 0.001

*AUC* area under the curve, *CIN* contrast-induced nephropathy, *NPV* negative predictive value, *PPV* positive predictive value, *Sens.* sensitivity, *Spec.* specificity

## Discussion

In this prospective cohort study, 500 STEMI patients free from prior history of significant kidney disease were recruited to evaluate the utility of CHA_**2**_DS_**2**_VAS**c** score in predicting CIN after pPCI. CHA_**2**_DS_**2**_VAS**c** score calculated at patients’ presentation proved to have excellent performance in CIN prediction. CHA_**2**_DS_**2**_VAS**c** proved to have similar overall accuracy compared to Mehran’s score derived after procedure completion and obtaining the results of the laboratory workup.

CIN remains as one of the most ominous adverse events for patients receiving parenteral iodinated contrast media for diagnostic or therapeutic procedures [1]. It is ranked the third most important cause of hospital-acquired AKI coming after hypoperfusion (or pre-renal injury) and postoperative AKI [20–22]. Despite increased awareness and continuous efforts for prevention, CIN or contrast-induced acute kidney injury (CI-AKI) remains a distressing cause of increased morbidity, longer hospital stays, 30-days and 1-year mortality [1, 4].

Average incidence of CIN for patients undergoing coronary angiography or PCI varied between registries from < 1% to 24% [4, 20, 23]. Such a wide range between different reports is probably attributed to different generations of iodinated contrast media, dissimilarities in the diagnostic definitions and most importantly, varying characteristics of the studied population. For elaboration, CIN incidence was as low as < 5% in populations with normal kidney functions, while despite adequate hydration, it reached 50% in another study selectively recruiting advanced diabetic nephropathy patients undergoing coronary angiography [24, 25]. Other features like patients’ age, anemia, diabetes and hypertension were also found to be of significant relevance to CIN occurrence [4, 7].

The underlying mechanism behind CIN is not fully understood; however, many contributing pathologies are incriminated. The excretion of the iodinated contrast media into the renal tubules leads to a sharp rise in the osmotic force, obligating the excretion of large amounts of water and solutes. This leads to a significant rise in the intratubular pressure, paralleled by proportionate decline in the GFR [2]. Simultaneously, exposure to iodinated contrast media causes a significant increase in the vasoconstrictor agents like endothelin and adenosine, and a significant decline in the vasodilatory effects of nitric oxide and prostaglandins [26]. All the above-mentioned pathologies induce intense hypoxia to the renal medulla and stimulate tubular cell apoptosis and death [2, 27, 28]. Liberation of oxygen free radicals also is believed to have a critical role in the pathogenesis of CIN [29].

Beside the incompletely understood pathogenesis, till the time being, we lack an effective dedicated therapy for such a serious condition [1, 30]. Moreover, the diagnosis of CIN is dependent on achieving the predefined rise in serum creatinine, which often starts to occur after 24 to 48 h from contrast exposure and peaks between the second to the fifth day of exposure [1]. This clearly means that the diagnosis of CIN is usually late because it depends on a delayed marker for AKI [4]. Despite having other more prompt and sensitive markers for AKI, their role as diagnostic tools for CIN is still investigational, while the general agreement for CIN diagnosis remains dependent on serum creatinine [31, 32].

Certainly, great efforts for primary prevention are required for such a serious condition with no specific treatment and with inherently delayed diagnosis. First in priorities, comes the early identification of patients at higher risk, aiming to timely address any correctable risk factors, to minimize the hazards and to improve clinical outcomes. Accordingly, many models were developed to quantify individual patient’s risk of CIN after iodinated contrast exposure.

Bartholomew et al. developed a risk stratification model based on > 20,000 patients undergoing PCI [7]. The risk score was an 8-points model with a score of 2 for each of: (1) creatinine clearance less than 60 mL/min, (2) use of intra-aortic balloon pump (IABP), (3) urgent/emergent PCI; and a score of 1 for each of: (4) diabetes, (v) hypertension, (6) congestive heart failure (CHF), (7) peripheral vascular disease and (8) contrast media volume (CMV) > 260 mL. In their cohort, none developed CIN in the low-risk group (score ≤ 1), while in the high-risk group (score of ≥ 9), CIN incidence reached 26% (*P*  < 0.0001). Despite the low general incidence of CIN in their cohort (2%), occurrence of CIN—(even without need for hemodialysis)—was associated with > 15-fold increase in rates of major adverse cardiac events, (MACE) [7].

Brown et al. [33] believed that the 2 main determinants for CIN occurrence are the CMV and individual patient’s baseline renal function represented by eGFR. They proposed a model for maximum allowable contrast dose (MACD) based on > 10,000 consecutive patients undergoing PCI with exclusion of those already requiring dialysis. MACD was deduced as (5 mL × body weight [in kg])/baseline serum creatinine [in mg/dL]. CIN occurrence was low and comparable between those receiving MACD and those receiving < MACD (*P* > 0.5). However, there was a proportionately increasing rate of CIN in those receiving 1.0 to 1.5, 1.5 to 2.0 and > 2.0 MACD with an adjusted OR (95% CI) of 1.6 (1.29–1.97), 2.02 (1.45–2.81) and 1.93 (1.93–4.48), respectively [33].

Contrarily, Ehramnn et al. [30] in a very well conducted systematic review and meta-analysis argued that AKI post-contrast exposure may be over-rated. According to their reasoning, the sole contribution of contrast media exposure to the resultant AKI is difficult to prove without large, controlled trials randomizing patients with similar clinical status to either receive iodinated contrast or not, which is practically implausible. Through their meticulous analysis of studies recruiting intensive care- and critically ill patients, they concluded that the rate of AKI was comparable between those receiving iodinated contrast media or not. They demonstrated that the independent added risk of contrast media exposure is at the most, very small, while the main determinant is the patients’ clinical profile [30].

Nevertheless, in the current practice, Mehran’s risk model is among the most accepted tools for prediction of CIN after PCI [5]. Mehran’s model was developed through studying > 8000 PCI patients, and is composed of 8 variables with different weights. A score of 5 is given to each of (1) hypotension, (2) use of IABP and (3) CHF; a score of 4 for (4) Age > 75 years; and a score of 3 to each of (5) anemia and (6) diabetes; (7) plus 1 point for each 100 mL of iodinated contrast; and (8) a score of 2, 4, or 6 according to eGFR if it was < 60, < 40 or < 20 mL/min/1.73m^2^, respectively [5].

Despite the wide agreement and extensive validation, systematic use of Mehran’s score on daily basis (outside clinical trials) is significantly limited. Complexity, difficult to memorize and inability to derive by the attending physician without a calculator or an aiding software, rendered the use of Mehran’s score very selective, triggered when clinically appreciating a high risk for CIN.

Considering STEMI patients, expediting reperfusion is a priority to maximize myocardial salvage and minimize rates of post-MI adverse sequelae, particularly in populations with ≥ 20% of STEMI patients are in the middle age active family earners [34]. Once STEMI diagnosis is established, the contemporary evidence prioritizes prompt reperfusion by pPCI over any delays (for echocardiography, cardiac troponin or any routine laboratory workup including kidney function tests) [6].

Additionally, compared to patients undergoing elective PCI, STEMI patients are more likely to be deprived from oral hydration through the preceding hours (thus, volume depleted), have higher rates of acute heart failure, cardiogenic shock and need for IABP, while have no time opportunity to allow for pre-procedural hydration, putting them at an inherently elevated CIN risk.

Despite the great similarity with Bartholomew’s [7], Mehran’s risk score [5] did not value the difference between elective and primary PCI procedures appreciated by the former [7], while actually “PCI for acute MI” was an exclusion criterion in the cohorts used for its development [5]. Also, its calculation will almost always be completed after the procedure ends, to integrate the laboratory results and the contrast volume. Hence, in the context of STEMI, chances that Mehran’s score offers a timely guidance to technical/procedural decisions are indefinite. On the other hand, CHA_**2**_DS_**2**_VAS**c** score represents one of the simplest stratification tools in medical practice. The abbreviation is quite memorizable and dictates swift calculation without any aids. Additionally, CHA_**2**_DS_**2**_VAS**c** only depends on personal and basic clinical features.

In this study of STEMI patients, distribution of age, diabetes, hypertension, heart failure, prior stroke/TIA, CMV, eGFR and the CMV/eGFR showed significant association with CIN, matching the evidence derived from prior studies and risk models [1, 3, 7, 33, 35]. Interestingly, among these variables, those which can be instantly elicited and are not dependent on time-consuming laboratory work represent the main body of CHA_**2**_DS_**2**_VAS**c** score. Furthermore, these very basic clinical features proved to be significant predictors for CIN in regression analysis.

Although originally developed for evaluation of thromboembolic risk in non-valvular AF [18, 19], CHA_**2**_DS_**2**_VAS**c** score comprehensively covers CV disease markers, comprising heart failure, hypertension, advanced age, diabetes, stroke, systemic embolization and established vascular disease. This greatly explains its universal predictive performance for adverse events noted in many other CV scenarios. In many studies in ACS context, CHA_**2**_DS_**2**_VAS**c** proved to be a sensitive predictor for new-onset AF [36], showed excellent performance in predicting stroke for those with and without AF [9], and proved to be an independent predictor of MACE (CV death, non-fatal MI, stroke) in another large study recruiting patients without AF [37]. In a large CKD cohort, CHA_**2**_DS_**2**_VAS**c** proved to be an independent predictor for CV mortality independent from atrial fibrillation [11]. Moreover, a high CHA_**2**_DS_**2**_VAS**c** score was a predictor for slow-flow and suboptimal-PCI-outcomes in both NSTEMI and STEMI patients [38, 39].

Regarding post-PCI CIN, in 2 large studies [40, 41] recruiting acute coronary syndrome (ACS) patients, CHA_**2**_DS_**2**_VASc proved to be an independent predictor for CIN occurrence, nevertheless, non-ST-segment elevation ACS (NSTE-ACS) comprised 61% and 37% of their recruited cohorts [40, 41].

Apart from the small-subset at very high risk, the majority of NSTE-ACS cases have adequate pre-PCI time to evaluate their kidney functions, quantify their CIN risk, plan staging of their revascularization and provide pre-procedure intravenous hydration as indicated, aiming to minimize their potential risks [8, 42]. Hence, STEMI patients are ideally looked and judged separately as a distinct category when concerning chances of pre-PCI evaluation and/or manipulation of their CIN risk.

Arguing against that CHA_2_DS_2_VASc is blinded to CMV and eGFR which are serious parameters in CIN risk is certainly a pertinent argument, but probably in settings different than STEMI when an informative risk indicator is critically needed before these data are made available. In other words, the comparable predictive accuracy of the promptly calculated CHA_2_DS_2_VASc score to the Mehran’s score calculated in hindsight can be point of strength in favor of the former.

Additionally, although CMV was a significant predictor in univariate analysis, it was not an independent predictor in multivariate regression, except when indexed by the eGFR. This goes in-lieu with the meta-analysis of Ehrmann et al. [30], who postulated that the sole impact of contrast exposure is at-the-most small, while the risk of AKI is essentially determined by the patient clinical background [30].

### Limitations

This study has some limitations. Exclusion of patients with history of prior significant renal dysfunction or previously or currently requiring hemodialysis made the study not representative to all STEMI patients, however, because these subsets are at distinct risk for post-PCI AKI that qualifies evaluation in a dedicated study. Similarly, was the exclusion of patients with cardiogenic shock whom the investigators perceived complexity in consenting for participation. Also, in many patients, creatinine was monitored for only 2 days and then patients with uneventful course were discharged, with a potential chance of missing CIN cases with late onset; nevertheless, this is presumably minimal because any patient with trend of increase in serum creatinine was exceptionally monitored more frequently and for longer than 2 days post-procedure.

### Clinical perspectives

Compared to elective PCI, STEMI patients undergoing pPCI are at increased risk of CIN and often have no opportunity to implement preventive measures.

Routine assessment of Mehran’s is significantly underutilized before pPCI, because of complexity and relying on timely unavailable components (hematocrit, eGFR and CMV).

CHA_2_DS_2_VASc score had excellent prediction for post-pPCI CIN comparable to Mehran’s score, with the advantage of being simple and completely informative before the procedure.

Adding systematic CHA_2_DS_2_VASc calculation in STEMI admission checklist can be very useful in guidance of intra- and postprocedural management to reduce CIN risk.

## Conclusions

For STEMI patients with no history of significant renal dysfunction, CHA_2_DS_2_VASc score calculated at presentation was an excellent predictor for post-pPCI CIN. CHA_2_DS_2_VASc ≥ 4 had comparable predictive accuracy to Mehran’s score ≥ 7, with the advantage of being timely informative before proceeding to the procedure. The beneficial impact of adding systematic calculation of CHA_2_DS_2_VASc score into STEMI admission checklists to guide procedural decisions is to be evaluated in future studies.

## Data Availability

Data can be provided (anonymized) upon reasonable request from the corresponding author.

## Abbreviations

ACS: Acute coronary syndrome
AKI: Acute kidney injury
ASCVD: Atherosclerotic cardiovascular disease
CHA_2_DS_2_VASC: Congestive heart failure, hypertension, age ≥ 75 (doubled), diabetes, stroke (doubled), vascular disease, age 65–4 and sex category (female)
CIN: Contrast-induced nephropathy
CKD: Chronic kidney disease
CMV: Contrast media volume
DES: Drug eluting stents
ECG: Electrocardiography
eGFR: Estimated glomerular filtration rate
HDL-C: High-density lipoprotein cholesterol
IABP: Intra-aortic balloon pump
IRA: Infarct-related artery
LDL-C: Low-density lipoprotein cholesterol
LVEF: Left ventricular ejection fraction
MACD: Maximum allowable contrast dose
MACE: Major adverse cardiac events
MDRD: Modification of diet in renal diseases
NSTE-ACS: Non-ST-segment elevation ACS
OR: Odds ratio
PCI: Percutaneous coronary intervention
pPCI: Primary percutaneous coronary intervention
STEMI: ST-elevation myocardial infarction
TIA: Transient ischemic attack
TIMI: Thrombolysis in myocardial infarction
